# Minimal residual disease negativity by next‐generation flow in non‐CR myeloma patients

**DOI:** 10.1002/jha2.103

**Published:** 2020-09-22

**Authors:** Sung‐Min Kim, Naery Euphrasia Yang, Dajeong Jeong, Jiwon Yun, Sohee Ryu, Sung‐Soo Yoon, Yong‐Oon Ahn, Dong Soon Lee

**Affiliations:** ^1^ Cancer Research Institute Seoul National University College of Medicine Seoul South Korea; ^2^ Department of Laboratory Medicine Sejong General Hospital Bucheon Republic of Korea; ^3^ Department of Laboratory Medicine Seoul National University Hospital Seoul South Korea; ^4^ Department of Internal Medicine Seoul National University College of Medicine Seoul South Korea; ^5^ Department of Laboratory Medicine Seoul National University College of Medicine Seoul South Korea

**Keywords:** MRD (minimal residual disease), Myeloma, NGF (next‐generation flow)

## Abstract

Next‐generation flow (NGF) has detected minimal residual disease (MRD) in numerous myeloma patients who achieve a complete response (CR). However, when MRD is not detected via NGF in non‐CR patients, its clinical meaning is uncertain. Here, we investigated the correlation between MRD findings on NGF and the response criteria, paying special attention to patients with discrepant results. We performed NGS analysis of IgH rearrangements on bone marrow samples from the non‐CR patients with negative MRD on NGF. NGS detected residual abnormal clones in those patients, suggesting that NGF and NGS should be used in a complementary manner for MRD investigation.

In multiple myeloma (MM), minimal residual disease (MRD) negativity has been shown to have prognostic significance [1, 2], surpassing the prognostic value of complete response (CR) [3]. At the same time, relapse rates remain high [4] despite improvements in treatments, thus necessitating a more in‐depth evaluation of patients with highly sensitive methods of MRD detection.

Next‐generation flow (NGF) and next‐generation sequencing (NGS) analyses have been adopted as additional bone marrow (BM) assessment tools for detecting MRD in the IMWG response criteria. While conventional flow cytometry has some limitations, the NGF technology enables the detection of several million cells, and the EuroFlow Consortium has provided fine‐tuned, standardized algorithms for identifying clonal PCs. NGF has detected MRD in many MM patients who achieved and remained at CR. These results are meaningful in that closer monitoring could benefit MRD‐positive patients with CR. However, when NGF fails to detect MRD in non‐CR patients, it is difficult to account for this discrepant phenomenon and its clinical meanings.

To address this issue, we investigated the correlation between NGF‐based MRD results and the IMWG response criteria and the biological implications of NGF in non‐CR patients, paying special attention to patients with discrepant results.

Thirty‐four myeloma patients under treatment were enrolled. With the follow‐up BM samples from these patients, NGF was carried out with eight‐color antibody panel. At the time of BM sampling for NGF, 11 patients achieved CR, 21 failed to reach CR, and two were not evaluable for treatment response. Four patients failed to achieve CR but showed MRD negativity by NGF. For those four patients, the NGS analysis of IgH rearrangements was conducted with paired BM specimens obtained at diagnosis and follow‐up evaluation (Figure 1).

**FIGURE 1:**
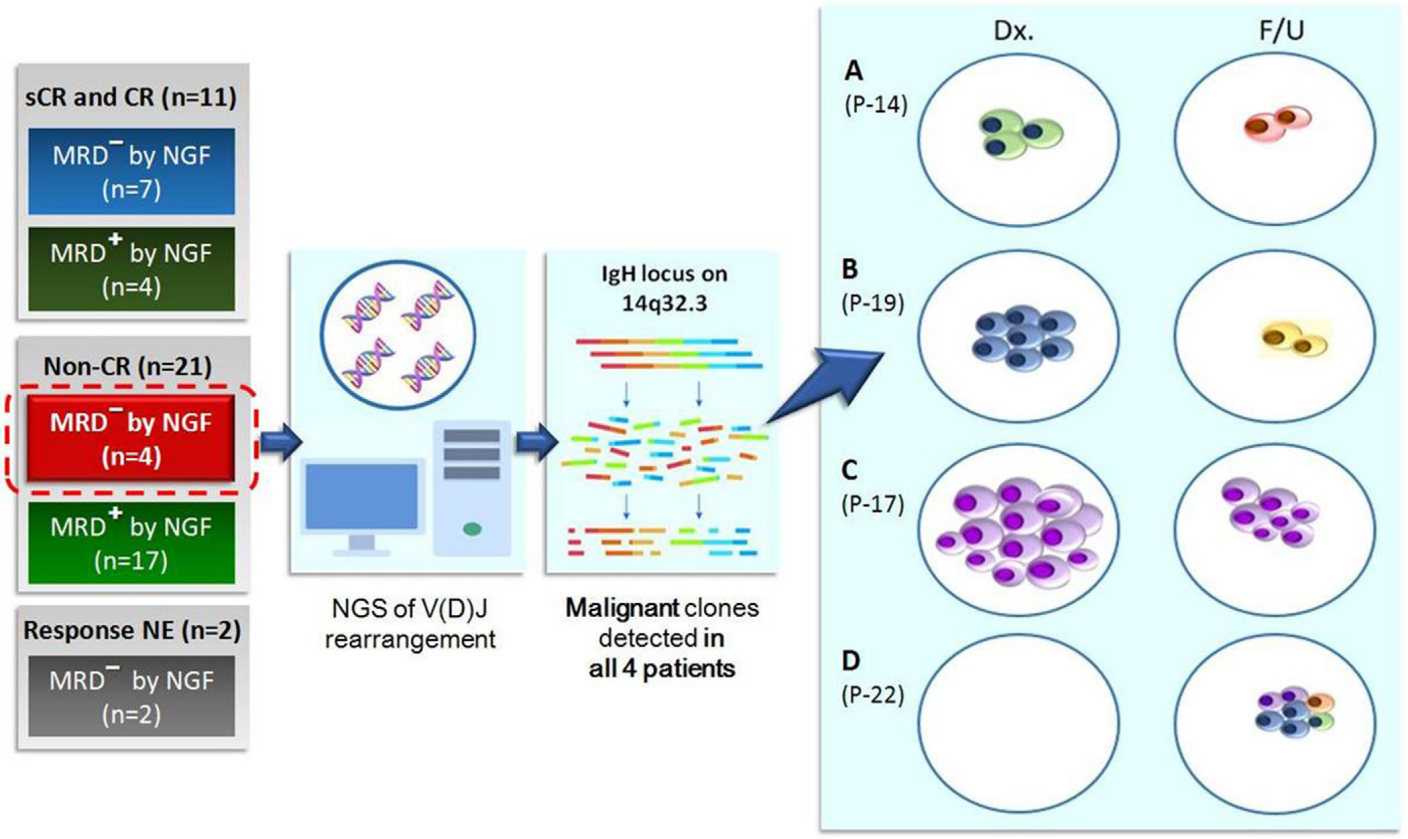
**NGS of IgH Rearrangements in the 4 non‐CR patients with negative MRD by NGF**. A and B, Acquisition of new dominant clones in two patients (P‐14 and P‐19). C, Persistence of the residual clone in one patients (P‐17). D, Acquisition of new heterogeneous clones in one patient (P‐22). CR, complete response; F/U, follow‐up; MM, multiple myeloma; MRD, minimal residual disease; NE, not evaluable; NGF, next‐generation flow; NGS, next‐generation sequencing; sCR, stringent complete response

NGF was performed based on the protocol of the EuroFlow Consortium [5, 6]. Bone marrow (BM) aspirates and peripheral blood samples were processed within 24 hours of sampling and incubated after RBC lysis (BulkLysis buffer; Cytognos, Salamanca, Spain) in two tubes (tubes 1 and 2) containing anti‐CD38 (FITC; Cytognos), anti‐CD56 (PE; Cytognos), anti‐CD45 (PerCP‐Cy5.5; BioLegend, CA, USA), anti‐CD19 (PE‐Cy7; Beckmann Coulter, FL, USA), anti‐CD117 (APC; BD Biosciences, CA, USA), anti‐CD81 (APC‐C750; Cytognos), anti‐CD27 (BV510; BioLegend), anti‐CD138 (BV421; BD Biosciences), anti‐CyIgκ (APC; Dako, CA, USA), and anti‐CyIgλ (Cytognos) antibodies. Both tubes containing up to 6 million cells each were subjected to RBC lysis and stained for surface markers, followed by incubation for 30 minutes at room temperature. Tube 2 was subjected to additional steps for intracellular staining for 15 minutes at room temperature. Up to 6 million events from each tube were acquired using a Navios flow cytometer (Beckmann Coulter). All instrument settings and compensation followed the EuroFlow standard operating protocol [7]. Data were analyzed using Infinicyt version 1.8 software (Cytognos).

For NGS, genomic DNA was prepared from BM aspirates using the QIAamp Blood Mini Kit (Qiagen, CA, USA) according to the manufacturer's instructions. DNA samples were submitted to Adaptive Biotechnologies (Seattle, WA, USA) for sequencing using the ImmunoSEQ IgH assay according to the previously published data [8]. Data were analyzed using the immunoSEQ Analyser toolset.

Of 34 patients, 21 showed abnormal PCs above the lower limit of quantification (LLOQ > 50 PC) on NGF. Among the 11 patients who achieved CR or stringent complete response (sCR), seven patients (63·3%) presented NGF‐MRD negativity. Of the 21 non‐CR patients, residual PCs were detected via NGF in 17 patients (81·0%). The remaining four non‐CR patients (19·0%), however, showed MRD negativity on NGF: one with a very good partial response, one with a partial response (PR), one with a minimal response, and one with stable disease (SD). To ensure that peripheral blood contamination did not lead to false negative NGF findings, we assessed the percentage of mast cells in the BM samples. We obtained a mast cell percentage of ≥0·002% in BM samples from all four patients, which was designated as the lowest mast cell percentage in non‐diluted BM [6], indicating that these samples were not diluted with blood (Table S1).

A minimum number of 2 million cells per tube were acquired for all of the samples in our study. Specifically, for the samples of the four non‐CR patients with negative MRD results by NGF, more than 5 million cells per tube were acquired. To remove batch effects, the NGF process for all of samples in this study was performed by one highly trained personnel, in precise accordance with the protocol of the EuroFlow Consortium.

The NGS study of IgH rearrangements on those four patients revealed residual abnormal PCs, which were not detected by NGF. The patient with PR (P‐17) harbored the same dominant clone in both the diagnostic BM (87·13%; proportion of clone) and the follow‐up BM (19·38%) samples. Two patients (P‐14 and P‐19) acquired new dominant clones after treatment, while the dominant IgH rearrangement clone detected in the diagnostic BM disappeared in each patient. The newly acquired dominant clone in P‐19 carried a nonproductive DJ rearrangement, whereas dominant clones found in the other three patients (P‐14, P‐17, and P‐22) harbored productive VDJ rearrangements. The patient with SD (P‐22) displayed heterogeneous clones in the follow‐up BM (5·24%, 4·72%, 3·11%, 2·09%), while no IgH rearrangements were detected in the diagnostic BM. The results of NGS of IgH rearrangements are summarized in Table 1.

**TABLE 1:** Results of IgH rearrangement NGS and main laboratory tests in the non‐CR patients with negative MRD on NGF

Case No.		P‐14	P‐17	P‐19	P‐22
IMWG treatment response		VGPR	PR	MR	SD
NGF MRD (%)		0·00	0·00017^a^	0·00	0·00
IgH rearrangement NGS (%)	Diagnosis	4·19	87·13	50·80	Not detected
	Follow‐up	0·75^b^	19·38	1·49^b^	5·24^b^ 4·72^b^ 3·11^b^ 2·09^b^
M‐protein serum (g/dL)		Not detected	0·23	0·13	2·08
sFLC κ/λ ratio		1·61 (normal)	1·98 (abnormal)	2·34 (abnormal)	11·36 (abnormal)

Abbreviations: IFE, immunofixation; IMWG, International Myeloma Working Group; MR, minimal response; NGF, next‐generation flow; NGS next‐generation sequencing; PR, partial response; SD, stable disease; sFLC, serum‐free light chain; VGPR, very good partial response.

^a^ Detected under limit of detection.

^b^ Acquisition of new dominant clones with disappearance of initial dominant clones at diagnosis.

The clinical impact of MRD negativity cannot be exaggerated in MM. Changes in the immunophenotype after treatment are not infrequent in MM, thus potentially yielding false negative MRD results on flow cytometry. The current EuroFlow NGF method, however, provides meticulous, sequential steps for yielding the highest resolution between normal and abnormal PCs [6], even if the immunophenotype is altered. Some studies have accounted for MRD‐negativity in non‐CR patients by the nature of M‐protein, which is more inert and has a longer half‐life; hence, M‐protein levels may not decrease promptly in response to treatment [9]. Normally, most serum proteins that are too large for renal filtration are cleared away through pinocytosis, which occurs in almost all nucleated cells. IgG has a concentration‐dependent half‐life of approximately 3 weeks because of the recycling process via FcRn receptors [10, 11, 12]. Furthermore, the IMWG criteria of treatment response primarily depend on the M‐protein and light chain concentrations; however, they do not consider the BM PC%, except that sCR and CR require a BM PC% <5. In other words, residual M‐proteins, which are cleared slowly, can lead to misclassification of virtual CR as non‐CR.

Antigenic drift of IgH rearrangements is frequent in B lymphoid malignancies, but NGS‐MRD measurement in myeloma has overcome these variations. In patients receiving treatment, we observe clonal antigenic evolution during the persistence of the residual clone and the emergence of new dominant and heterogeneous clones. However, an interpretation for dominant clones is not yet standardized for NGS. The criteria for defining dominant sequences are rather arbitrary. A commercial NGS service provider, Clonoseq, has defined a dominant sequence as those comprising at least 3% of all similar sequences in sequences among IgH, IgK, and IgL for diagnostic purposes. Some studies adopted 0·3‐0·5% [13, 14] as the threshold for a dominant clone. In our study, the threshold for dominant sequences was set at 0·7%, and NGS of IgH rearrangement revealed dominant neoplastic sequences in all four non‐CR patients with negative MRD on NGF. This suggests that the BM samples were neither diluted nor inadequate for evaluation. The percentage of mast cells in BM samples further indicated that peripheral blood contamination was an unlikely explanation for MRD negativity on NGF.

The present study shows that NGS can be used to detect residual clones in patients who test MRD‐negative by NGF. We suggest that NGF and NGS should be performed in a complementary manner to determine the MRD status. We also suggest that once one method (NGF or NGS) yields negative results, the other be applied to assess the validity of the negative finding. Therefore, NGF and NGS can compensate for the deficits in the IMWG treatment response criteria, which are mainly based on M‐protein levels. The IMWG suggests that MRD tests be initiated only at suspected CR. However, since the prognostic value of MRD negativity has been proven to surpass CR in studies [1, 3], it is necessary for all patients to be screened for the MRD status regardless of their response criteria. In addition, whether non‐CR patients with MRD negativity on NGF have better outcomes than MRD‐positive patients within the same response groups warrants further investigation.

## CONFLICT OF INTEREST

The authors declared that there is no conflict of interest.

## AUTHOR CONTRIBUTIONS

D.S.L. designed the research study and interpreted the data; S.M.K. and N.E.Y. performed most of the work under the planning and guidance of D.S.L.; S.S.Y. was involved in the clinical care of the patients; D.S.L., S.M.K., D.J., J.Y., S.R., S.S.Y., and Y.O.A. reviewed the manuscript and edited the manuscripts created by N.E.Y and S.M.K.

## Supporting information


**Table S1**. Summary of next‐generation flow study.

## Data Availability

The data that support the findings of this study are available from the corresponding author upon reasonable request.
