# Supplementary material for: Minimal residual disease negativity by next‐generation flow in non‐CR myeloma patients
Source: EJHaem. 2020 Sep 22;1(2):563–6. doi: 10.1002/jha2.103 (PMC9175670; doi:10.1002/jha2.103)
Supplement: Supplementary file 1 — Table S1. Summary of next‐generation flow study. [file JHA2-1-563-s001.docx]

**Supplemental Table SI. Summary of Next-generation Flow study.**

| Number of samples analyzed | 34 |
| --- | --- |
| Total acquisition No. of cells,  Median (Range) | 10,119,230  (6,195,271 – 11, 339, 302) |
| No. of samples  with total acquisition No. of cells >5 x 10^6^ | 34 |
| No. of samples with >85% of viability | 33 |
| No. of patients with abnormal PCs  below LOD (20 events) | 10 |
| No. of patients with abnormal PCs  between LOD and LLOQ | 3 |
| No. of patients with abnormal PCs  above LLOQ (50 events) | 21 |
| Median (Range) % of MRD  in samples with abnormal PCs above LLOQ | 0·0571%  (0·0005% – 29·6856%) |

MRD, minimal residual disease; LOD, limit of detection; LLOQ, lower limits of quantification; PCs, plasma cells.
